# Distinct EEG functional network alterations in Parkinson's disease and multiple system atrophy

**DOI:** 10.1016/j.nicl.2026.104072

**Published:** 2026-09-27

**Authors:** Daichao Chen, Huada Tang, Yanfei Pan, Zhuangzhuang Zhang, Wenzhe Wang, Fangda Leng, Haotian Yan, Hui Sang, Kai Li, Fan Li, Yunchuang Sun, Jing Chen, Wei Sun, Jue Zhang, Zhaoxia Wang, Luhua Wei

**Affiliations:** a Department of Neurology, Peking University First Hospital, Beijing 100034, China; b Academy for Advanced Interdisciplinary Studies, Peking University, Beijing 100871, China; c Fein Memory and Aging Center, Department of Neurology, University of California, San Francisco, CA 94158, USA; d Department of Neurology, Xuanwu Hospital, Capital Medical University, Beijing 100053, China.; e Department of Neurology, Taiyuan City Central Hospital, Taiyuan 030009, Shanxi, China; f School of Mechanics and Engineering Science, Peking University, Beijing 100871, China

**Keywords:** Electrophysiology, Functional connectivity, Cortical networks, Neural dynamics, Machine learning, Parkinson's disease, Multiple system atrophy

## Abstract

Parkinson's disease (PD) and multiple system atrophy (MSA) are alpha-synuclein-related neurodegenerative disorders with overlapping motor and non-motor symptoms but distinct pathology, clinical management, and prognosis. Early differential diagnosis, particularly between PD and the parkinsonian subtype of MSA (MSA-P), remains a major clinical challenge. Yet, how their distinct underlying neurodegeneration differentially disrupts large-scale neural coordination remains poorly understood, limiting the development of objective neuroimaging biomarkers. Here, we aimed to characterize EEG cortical network alterations in PD and MSA subtypes and explore the potential discriminative value of these features. Using a data-driven masking empirical mode decomposition approach, we isolated cortical oscillatory modes from 97 participants (28 PD, 42 MSA, 27 healthy controls) and computed amplitude- and phase-based functional connectivity. Exploratory network-based statistics identified divergent patterns of disruption, where MSA was characterized by β-band amplitude-based hypoconnectivity, while PD exhibited phase-based decoupling across the δ and β bands. While both disorders shared common network correlates for specific clinical impairments, including α-band phase decoupling for bradykinesia and slow-wave hyperconnectivity for non-motor decline, they also exhibited divergent network associations with symptom severity, highlighting that functional network alterations scale differently with clinical impairment in the two disorders. Finally, employing a target residual machine-learning framework, we conducted an exploratory internal-validation analysis indicating that integrating these EEG network features with standard clinical assessments provided a modest incremental gain in discrimination between PD and MSA-P. These findings highlight the value of source-level EEG functional connectivity in capturing disease-specific neural circuit disruptions, offering candidate features for future validation as objective biomarkers for the differential diagnosis and pathophysiological tracking of parkinsonian disorders.

## 1 Introduction

Parkinson's disease (PD) and multiple system atrophy (MSA) are alpha-synuclein-related neurodegenerative disorders with overlapping motor and non-motor symptoms, yet they demonstrate distinct pathophysiology, progression, and treatment responsiveness (Kalia and Lang, 2015; Krismer et al., 2024; Poewe et al., 2022). Clinically, distinguishing MSA, especially the parkinsonian subtype, from PD remains challenging due to overlapping parkinsonian features, particularly in the early stages (Palermo et al., 2020), as evidenced by several neuropathological validation studies (Koga et al., 2015; Virameteekul et al., 2023). Since the treatment approaches and prognoses of the two diseases differ significantly, developing novel objective biomarkers is highly desired for improving the differential diagnosis and management of PD and MSA (Leng et al., 2025; Luan et al., 2022; Zheng et al., 2022).

To address this diagnostic challenge, standard clinical assessments are increasingly supplemented by objective functional biomarkers. Classical clinical neurophysiological evaluations, such as electromyography (Cao et al., 2020), oculomotor tracking (Zhou et al., 2024), and brainstem reflex testing (Sciacca et al., 2020a, Sciacca et al., 2020b), have proven highly valuable by probing specific sensorimotor and brainstem circuits for differential diagnosis between PD and MSA. While these clinical neurophysiological tools provide excellent focal diagnostic markers, the widespread and divergent neurodegeneration in PD and MSA also profoundly disrupts large-scale neural coordination across the cortex. Capturing these broader, system-level abnormalities requires a macro-scale network approach.

To this end, functional brain imaging, such as functional MRI (fMRI) and EEG, offers powerful tools for investigating the underlying circuit dysfunction in neurodegenerative diseases. Given that neurodegeneration in PD and MSA inherently differs in affected cell types and pathological distribution, their neural circuits should exhibit distinct abnormalities in large-scale network dynamics. While fMRI studies in PD and MSA have revealed abnormal connectivity involving the default mode network (DMN) (Franciotti et al., 2015; Rosskopf et al., 2018) and the sensorimotor network (SMN) (Lyu et al., 2023; You et al., 2011), EEG offers distinct advantages for capturing dynamic, millisecond-scale oscillatory abnormalities that may better reflect disease-specific pathophysiology. Historically, EEG studies in these disorders have largely focused on spectral slowing and band power alterations (Barcelon et al., 2019; Geraedts et al., 2018; Jaramillo-Jimenez et al., 2023; Liu et al., 2023). While these power alterations confirm cortical involvement, they do not capture how communication between distributed brain regions is impaired. Functional connectivity (FC) offers a more comprehensive view of large-scale cortical circuit disruptions, as neural oscillations synchronize through both phase coordination and amplitude fluctuations. In PD, FC analysis has been extensively performed, showing hypoconnectivity subnetworks in the δ, θ, α, and β bands as well as hyperconnectivity in the β band; crucially, the strength of these altered connections correlates strongly with motor and non-motor symptoms, including bradykinesia, gait disorders, and cognitive impairment (Conti et al., 2022, Conti et al., 2023; Yassine et al., 2022; Zhang et al., 2022). In contrast, FC analysis in MSA is still in its infancy. An initial study found that MSA exhibits weaker phase-based coupling in the α-band, which correlates with motor symptom severity (Wang et al., 2024). Comparative studies available suggest divergent network alterations between the two disorders, with MSA demonstrating stronger coupling in the θ-band (Liu et al., 2023) and γ-band (Wang et al., 2024) compared to PD.

Despite the progress showing the potential of EEG to capture divergent circuit dysfunction in PD and MSA, a more detailed characterization of EEG functional network alterations in MSA, as well as their clinical relevance, remains largely unexplored and has not yet achieved satisfactory clinical utility (Suppa et al., 2025). Prior studies (Barcelon et al., 2019; Liu et al., 2023) mainly focused on electrode-level analysis, which is not enough for understanding neural network functions or developing reliable EEG biomarkers. Furthermore, these studies have not accounted for the heterogeneity between the parkinsonian (MSA-P) and cerebellar (MSA-C) subtypes, which likely possess distinct pathophysiological trajectories. Consequently, to understand how circuit disruptions produce clinical phenotypes, analysis must align with the brain's functional architecture, namely, its connected and specialized cortical networks that mediate distinct motor and non-motor functions. These include the dorsal attention network (DAN), the central executive network (CEN), the limbic network (LN), the visual network (VN), the salience network (SN), DMN, and SMN (Schaefer et al., 2018; Yeo et al., 2011). Disruptions in their intra- and inter-connection may underlie the divergent clinical phenotypes of PD, MSA-P, and MSA-C, making cortical source network-level analysis essential for investigating clinical relevance.

To this end, we utilized source-reconstructed resting-state EEG to characterize multiband functional network alterations in PD and MSA (Fig. 1). To accurately capture the non-linear and non-stationary dynamics of resting-state EEG, we utilized a data-driven empirical mode decomposition (EMD) approach to adaptively isolate intrinsic oscillatory modes lying in different frequency bands. We then examined both amplitude- and phase-based functional connectivity on cortical network nodes and linked these network signatures to key motor and autonomic symptoms to bridge cortical electrophysiology with the clinical heterogeneity of parkinsonism. Finally, we conducted an exploratory internal-validation analysis to evaluate the discriminative potential of these features using machine learning for differential diagnosis between PD and MSA-P. Together, our work provides a proof-of-concept demonstration that source-level EEG can reveal disease- and symptom-specific functional network fingerprints in PD and MSA, providing a better understanding of their circuit mechanism and highlight the potential of EEG network dynamics to inform the future differential diagnosis of parkinsonian disorders.

**Fig. 1:**
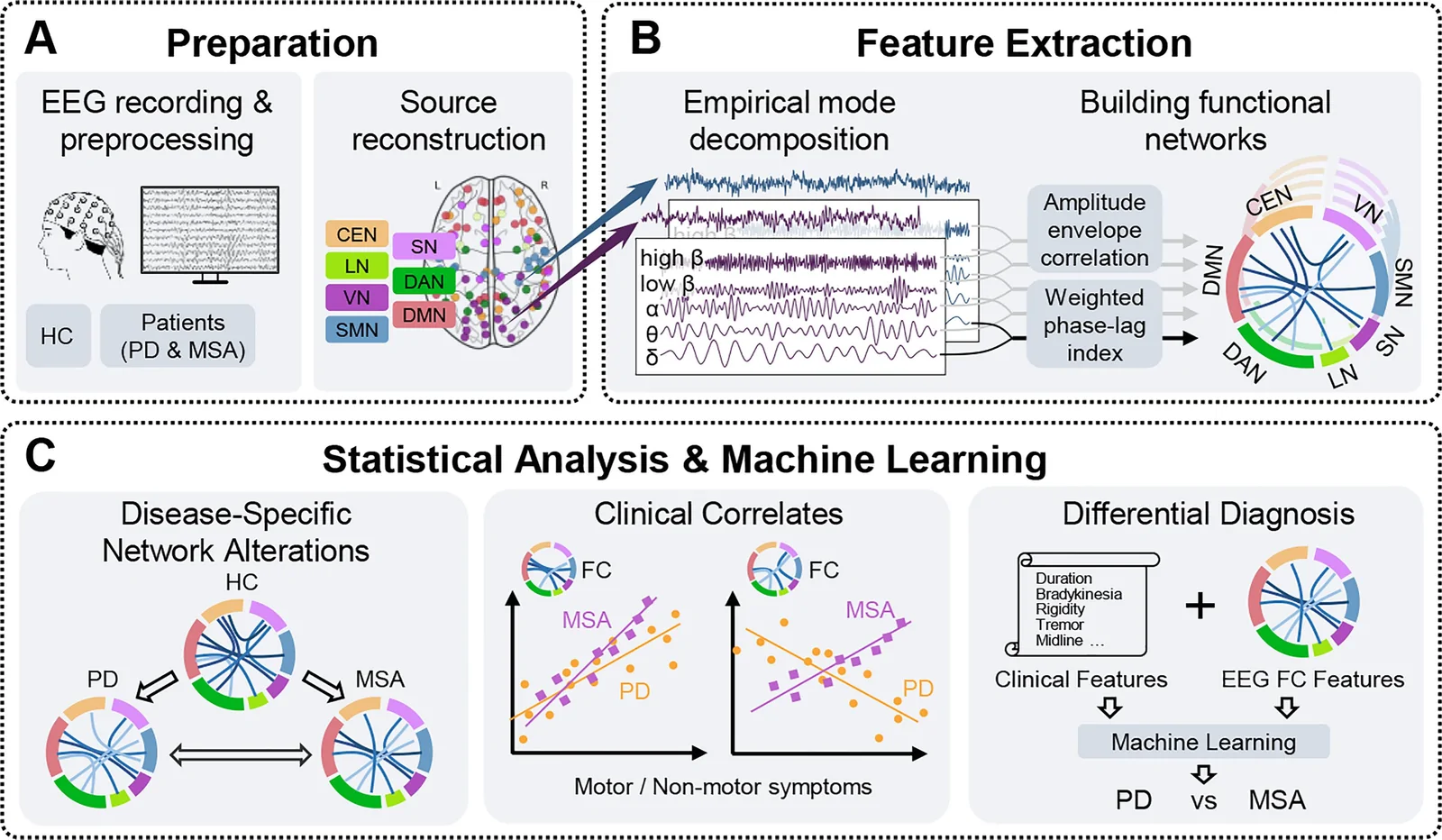
Schematic overview of the study design and analytical workflow. (A) Preparation. Resting-state EEG data were recorded from healthy controls (HC) and patients with Parkinson's disease (PD) and multiple system atrophy (MSA). Following standard preprocessing, sensor-level data underwent source reconstruction to map cortical activity across seven major resting-state networks, including the central executive network (CEN), the limbic network (LN), the visual network (VN), the sensorimotor network (SMN), the salience network (SN), the dorsal attention network (DAN), and the default mode network (DMN). (B) Feature extraction. Source-localized signals underwent empirical mode decomposition to isolate distinct frequency bands (δ, θ, α, low β, and high β). Functional networks were then constructed using two functional connectivity (FC) metrics, weighted phase-lag index and amplitude envelope correlation. (C) Statistical analysis and machine learning. Extracted network features were utilized for three primary analyses: (i) identifying disease-specific network alterations by comparing FC among HC, PD, and MSA groups; (ii) investigating clinical correlates to reveal shared and divergent brain-symptom relationships in PD and MSA; and (iii) developing a machine learning model using clinical scores and EEG FC features for differential diagnosis between PD and MSA.

## 2 Materials and methods

### 2.1 Participants

Participants with PD or MSA and healthy controls (HCs) were recruited at Peking University First Hospital between 2022 and 2026. Inclusion criteria for the patient groups were: 1) age 40–85 years; 2) clinically established or probable idiopathic PD according to the 2015 International Parkinson's and Movement Disorder Society (MDS) criteria (Postuma et al., 2015), and clinically established or probable MSA according to the 2022 MDS criteria (Wenning et al., 2022); 3) modified Hoehn-Yahr stage I–IV. Exclusion criteria included: 1) cognitive impairment (Chinese Mini Mental Status [CMMS] (Katzman et al., 1988) < 24 or Montreal Cognitive Assessment [MoCA] score < 21); 2) a history of epilepsy, stroke, or other severe neurological or psychiatric disorders; 3) severe resting tremor (defined as a score of 4 on any resting tremor sub-item of the MDS Unified Parkinson's Disease Rating Scale part III [MDS-UPDRS III]) to minimize movement artifacts during EEG acquisition. All participants provided written informed consent prior to clinical assessment and EEG recording. Only participants with EEG recordings of sufficient technical quality were included in the final analysis. Patient diagnoses were confirmed by two experienced neurologists. MSA was subtyped into the parkinsonian (MSA-P) or the cerebellar (MSA-C) subtype based on whether parkinsonism or cerebellar ataxia was the predominant motor manifestation, following established criteria (Wenning et al., 2022). Because early-stage parkinsonian disorders can be difficult to differentiate, diagnoses for patients with ambiguous initial presentations were confirmed longitudinally through subsequent follow-ups.

Motor symptoms of these patients were assessed by MDS-UPDRS III and Unified Multiple System Atrophy Rating Scale Part II (UMSARS II, only for MSA patients) immediately following EEG collection. Non-motor symptoms were assessed by Non-Motor Symptoms Questionnaire (NMSQ) within a 3-month window surrounding the EEG collection. For all clinical scales utilized in this study, higher scores indicate greater symptom severity. The study was approved by the Peking University First Hospital Ethics Committee.

### 2.2 EEG data acquisition and preprocessing

All participants underwent 3-min resting-state EEG recordings in the eye-closed condition. Data were acquired using either the Neurofax EEG-1200 system (Nihon Kohden, Tokyo, Japan; *n* = 50) or the PN-NET system (Yunshen Technology, Beijing, China; *n* = 47) in combination with a Greentek 64-channel cap conforming to the 10–10 international system. The distribution of the two EEG devices did not differ significantly across the groups (15 / 12 in HC, 16 / 12 in PD, 10 / 9 in MSA-P, and 9 / 14 in MSA-C, *P* = 0.582 using the Chi-squared test), reducing the risk of hardware-related confounding. Since residual hardware effects may remain, the recording device was included as a nuisance covariate in subsequent statistical analyses. Signals were digitized at a sampling rate of 1000 Hz, and electrode impedances were kept below 10 kΩ. Patients were recorded in their off-medication state. During the recording, participants were seated in a comfortable chair and instructed to remain awake, relax, and minimize body movements.

EEG preprocessing was implemented using custom Python scripts based on the MNE-Python (Gramfort et al., 2013) and the MEEGkit packages. Initially, channels and data segments exhibiting excessive noise or poor data quality were manually annotated and excluded. Data were downsampled to 256 Hz, and 50 Hz line noise was removed using the iterative Zapline method (implemented via the “dss_line_iter” function in MEEGkit, with win_siz = 8 and spot_siz = 3) (de Cheveigné, 2020). The data were then re-referenced to the common average reference, excluding the previously annotated bad channels. To further remove artifacts, independent component analysis (ICA) was applied, and components predicted by MNE-ICALabel (Li et al., 2022) as “eye”, “heart”, “muscle”, “line noise”, or “channel noise” with a probability ≥0.7 were rejected. Following ICA, signals were band-pass filtered between 1 and 80 Hz using a one-pass, zero-phase FIR filter (with a transition band of 1 Hz for low cutoff and 10 Hz for high cutoff). Previously removed bad channels were interpolated using spherical spline interpolation. Finally, the continuous signals were segmented into 8-s epochs with a 50% overlap, omitting the previously annotated bad time segments. Importantly, the data quality remained consistent across all cohorts; there were no significant differences among the PD, MSA, and HC groups regarding the number of interpolated channels (mean ± SD across all subjects: 4.4 ± 2.6; *P* = 0.992) or the number of rejected independent components (5.0 ± 3.7; *P* = 0.149) using non-parametric Kruskal-Wallis tests.

### 2.3 Source reconstruction

To estimate electrophysiological activity at the cortical level, source reconstruction was performed using the MNE-Python package (Gramfort et al., 2013). The cortical source space was defined on the FreeSurfer fsaverage template brain (Fischl et al., 1999) and comprised 8196 dipoles distributed using a recursively subdivided octahedron (oct6). Forward modeling was constructed using standard electrode positions and a three-layer boundary element model (comprising inner skull, outer skull, and skin) computed from the template.

To account for rank deficiency introduced during preprocessing, the data rank was explicitly estimated. The noise covariance matrix was then computed using the Oracle Approximating Shrinkage (OAS) method (Chen et al., 2010) to ensure a stable and well-conditioned matrix. The exact low-resolution brain electromagnetic tomography (eLORETA) algorithm (Pascual-Marqui et al., 2011) was utilized to invert the forward model. During the inverse operator computation, dipole orientations were loosely constrained (loose = 0.8), and final source estimates were restricted to the direction normal to the cortical surface. Regularization was applied based on a signal-to-noise ratio of 1.0, which is appropriate for resting-state EEG data.

Finally, the reconstructed cortical dipoles were parcellated into 100 regions of interest (ROIs) according to the 7-network version of the Schaefer atlas (Schaefer et al., 2018), where each ROI belongs to one of the seven cortical networks (DAN, CEN, LN, VN, SN, DMN, and SMN). To extract a single representative time course for each ROI, we applied a sign-flip averaging approach. This method aligns the polarity of the dipole time series within a given label to the dominant orientation prior to averaging, effectively preventing signal cancellation caused by the convoluted geometry of the cortex.

### 2.4 Empirical mode decomposition

To isolate the ROI time series into distinct frequency bands while accommodating the non-linear and non-stationary nature of EEG signals, we utilized Empirical Mode Decomposition (EMD) (Huang et al., 1998). Standard EMD relies on a data-driven sifting process to extract intrinsic mode functions (IMFs). However, signal intermittency can cause ‘mode mixing’ where low-frequency oscillations leak into higher-frequency IMFs; for instance, α oscillation is split between IMF4 and IMF5, and also leaking into IMF3 (Supplementary Fig. S1A, left). Furthermore, the purely data-driven nature of standard EMD makes it difficult to consistently align IMFs to canonical EEG frequency bands across different ROIs and subjects.

To resolve mode mixing and enforce cross-subject IMF alignment, we applied Masking EMD (Deering and Kaiser, 2005). This approach utilizes sinusoidal masking signals to prevent spectral leakage and guide the decomposition. Masking frequencies were set at 65, 25, 16, 10, 6, and 3 Hz, yielding six distinct components (IMF1–IMF6) and effectively reducing mode mixing (Supplementary Fig. S1A, right). The first component (IMF1) was discarded to exclude potential high-frequency noise and muscle artifacts. The center frequencies of the remaining five IMFs (IMF2–IMF6) aligned robustly with classical EEG frequency bands: high β (20–35 Hz), low β (13–20 Hz), α (8–13 Hz), θ (4–8 Hz), and δ (1–4 Hz), respectively (Supplementary Fig. S1B, C). These isolated IMFs, designated as high β-, low β-, α-, θ-, and δ-IMFs, were subsequently used to compute band-specific functional connectivity.

### 2.5 Functional connectivity

To assess large-scale neural coupling, we computed two complementary functional connectivity metrics, amplitude envelope correlation (AEC) (Hipp et al., 2012) and weighted phase-lag index (wPLI) (Vinck et al., 2011). These metrics were calculated for the δ-, θ-, α-, low β-, and high β-IMFs for each pair of ROIs.

Crucially, both metrics were selected or modified to mitigate the effects of spatial leakage (volume conduction) inherent in EEG source reconstruction. AEC measures the correlation of power envelopes to reflect slow, modulatory co-activation. We employed the orthogonalized approach (Hipp et al., 2012) on the analytical signals to remove zero-lag correlations. wPLI measures phase consistency and captures precise timing-based synchrony, which inherently suppresses interactions at 0 and 180 degrees, also making it robust to volume conduction.

AEC and wPLI were computed for each 8-s epoch, and then averaged across all epochs to obtain the final 100 × 100 AEC and wPLI functional connectivity matrices for each frequency band per subject. A Fisher's *r*-to-*z* transformation was applied to all AEC matrices to improve the distribution of the correlation coefficients values, making them closer to a normal distribution for subsequent general linear modeling.

### 2.6 Statistical analysis

#### 2.6.1 Network-based statistic

To identify significant subnetworks while controlling for the family-wise error rate (FWER) across the 100 × 100 connectivity matrices, we utilized the network-based statistic (NBS) framework (Zalesky et al., 2010). For all analyses, edge-level statistical evaluation was performed using General Linear Models (GLMs). An initial uncorrected primary threshold of *P* < 0.005 was applied to the edge-level *t*-statistics to define connected network components. The statistical significance of these components was then evaluated using non-parametric permutation testing (5000 permutations), yielding FWER-corrected *P* values. Statistical significance for the subnetworks was defined as within-analysis FWER-corrected *P* ≤ 0.05. It is important to note that while the NBS procedure controls for multiple comparisons across the edges within a given connectivity matrix, it does not inherently correct for the multiple separate analyses performed across different frequency bands, metrics, contrasts, and clinical scores. The NBS results presented here should be interpreted as exploratory. To accommodate nuisance covariates, permutations were performed using the Freedman-Lane procedure (Freedman and Lane, 1983; Winkler et al., 2014), subject to the exchangeability assumptions of the model. Briefly, this involved fitting a reduced model excluding the effect of interest, permuting the resulting residuals, and adding these shuffled residuals back to the reduced model's predictions prior to recalculating the GLM *t*-statistics. This approach ensures that the variance associated with nuisance covariates is rigorously controlled during the generation of the null distribution.

#### 2.6.2 General linear modeling strategy

Edge-level *t*-statistics within the NBS framework were derived using a stepwise GLM approach to evaluate group differences and clinical correlates. In all models, age, gender, and the recording device were included as nuisance covariates to ensure rigorous control over demographic and hardware-related confounding.

(1) Disease-specific network alterations: To identify divergent functional network topology across groups, group contrasts (HC vs. PD, HC vs. MSA-P, HC vs. MSA-C, and PD vs. MSA-P) were evaluated. The GLM predicted edge-wise connectivity as a linear combination of the group factor and nuisance covariates. Because patient groups inherently differed in disease progression profiles, we conducted an additional sensitivity analysis for the direct PD vs. MSA-P contrast, where Levodopa equivalent daily dose (LEDD), disease duration and MDS-UPDRS III total scores were included as additional continuous nuisance covariates. This allowed us to verify whether connectivity differences remained consistent after accounting for discrepancies in medication dosage, disease stage and motor impairment severity.
(2) Shared clinical correlates: To identify brain-symptom relationships shared between PD and MSA for motor and non-motor impairments (including MDS-UPDRS III sub-scores for bradykinesia, rigidity, and tremor, as well as NMSQ sub-scores for autonomic, psychiatric, and cognitive symptoms), we tested the main effect of each symptom severity. The GLM included the group factor, the clinical score, and nuisance covariates. Including the group factor as a covariate in this model was crucial to isolate the continuous effect of symptom severity from baseline categorical disease differences.
(3) Divergent clinical correlates: To investigate whether PD and MSA exhibit distinct clinical associations (i.e., disease-specific brain-behavior relationships), an interaction term was introduced. The GLM predicted connectivity using the group factor (coded as PD = 0, MSA = 1), the clinical score, the group × clinical score interaction, and nuisance covariates. Based on this dummy coding, a positive *t*-statistic for the interaction term indicates that the positive association between symptom severity and connectivity is significantly steeper in MSA compared to PD.

For visualization and descriptive purposes in analyzing clinical correlates, the mean functional connectivity of the significant subnetworks identified via NBS was extracted. Residuals were calculated by regressing out the nuisance covariates and diagnosis to plot partial correlations which demonstrates the group-specific clinical associations. NBS and GLM were implemented in Python based on the Scipy (Virtanen et al., 2020) and the NetworkX (Hagberg et al., 2008) packages.

### 2.7 Exploratory machine learning analysis

To explore the potential the diagnostic utility of EEG functional connectivity in differentiating PD from MSA-P, we developed an exploratory predictive model using a target residual learning framework (Supplementary Fig. S5). This approach was specifically designed to determine whether EEG features provide complementary diagnostic information beyond standard clinical assessments.

#### 2.7.1 Feature engineering and harmonization

To reduce dimensionality and prevent overfitting, the 100-ROI functional connectivity matrices were averaged into 14 major cortical networks (7 canonical networks across the left and right hemispheres). For each subject, the upper triangular and diagonal elements of these 14 × 14 matrices were extracted across all 5 frequency bands and 2 metrics (AEC and wPLI), resulting in a 1050-dimensional feature set representing all intra- and inter-network interactions. To mitigate hardware-related batch effects, we applied a device-specific harmonization strategy. The EEG features for the PD and MSA-P groups were standardized (z-scored) using the mean and variance derived exclusively from the out-of-sample groups (HC and MSA-C) recorded on the corresponding device. This approach harmonizes the feature space across devices while strictly preventing target-class data leakage prior to machine learning.

#### 2.7.2 Target residual learning framework

The classification framework consisted of two sequential stages. In the first stage, a base clinical model (*L*_2_-penalized logistic regression with balanced class weights) was trained on standard clinical assessments, including disease duration, modified Hoehn & Yahr scale, and MDS-UPDRS III total and sub-scores (total/left/right bradykinesia, rigidity, tremor; and total midline symptoms), to output a baseline probability of the true diagnosis. The “target residual” was then calculated as the difference between the true binary label and this clinically predicted probability. In the second stage, an EEG-specific model was trained to predict this residual error. To handle high dimensionality, univariate feature selection (based on F-statistics) was first applied to select the top 40 most informative EEG features correlated with the residual. A support vector regressor (with the radial basis function kernel) was then trained on these selected features. The final diagnostic probability was computed by adding the EEG-derived residual adjustment to the baseline clinical probability, capped between 0 and 1. The inverse regularization strength (*C*) for both the logistic regression and the support vector regressor was fixed at 0.1 to enforce strong *L*_2_ penalty.

#### 2.7.3 Validation and interpretability

Model performance was evaluated using repeated stratified K-fold cross-validation (8 splits, 100 repeats, yielding 800 folds). Crucially, feature selection and data scaling were strictly confined to the training data within each cross-validation iteration to prevent data leakage. Classification performance of the base model (trained on clinical features) and the combined model (trained on clinical features and EEG features) was quantified using accuracy, precision, sensitivity, specificity, and F1 score. To assess whether the EEG feature structure contributed meaningful diagnostic information, we performed an EEG-feature permutation test. Clinical variables and diagnostic labels were kept fixed, whereas EEG feature were randomly shuffled across subjects. For each permutation, the entire repeated cross-validation pipeline was rerun. The observed performance gain, defined by the difference of mean cross-validation performance between the combined model and the clinical model, was compared against the null distribution obtained from 1000 permutations.

Finally, to ensure the model remained interpretable, we employed Shapley additive explanations (SHAP) (Lundberg and Lee, 2017). SHAP values were calculated across all test folds to determine the exact contribution of specific clinical and EEG features to the final diagnostic prediction. It should be noted that SHAP does not perform hypothesis testing for statistical inference. Instead, it is a post-hoc model interpretability framework used strictly to understand the internal logic and feature weighting of the trained classifier.

## 3 Results

### 3.1 Participant characteristics

A total of 97 participants were included in the study, comprising 27 healthy controls (HC), 28 patients with PD, and 42 patients with MSA (19 MSA-P and 23 MSA-C), with their demographic and clinical characteristics summarized in Table 1. The four groups were comparable in terms of age (Kruskal-Wallis test, *P* = 0.353), distribution of gender (Chi-square test, *P* = 0.230), distribution of handedness (*P* = 0.897), and years of education (*P* = 0.167). Among the patient cohorts, significant differences were observed in disease duration (*P* = 0.001). Post hoc pairwise comparisons with false discovery rate (FDR) correction revealed that patients with PD had a longer disease duration compared to the MSA-C group (*P* = 0.001). Regarding motor impairment, the MSA-P group exhibited higher MDS-UPDRS III scores compared to both the PD and MSA-C groups (both *P* = 0.006), reflecting the more rapid and severe parkinsonian progression characteristic of MSA-P. The overall burden of non-motor symptoms (NMSQ) did not differ significantly across the three patient groups (*P* = 0.595). Regarding treatment exposure, LEDD differed significantly among the patient groups (*P* < 0.001). Post hoc comparisons revealed that the MSA-C group had significantly lower LEDD compared to both the PD and MSA-P groups (both *P* < 0.001), aligning with the cerebellar-predominant presentation and limited use of dopaminergic therapy in MSA-C. LEDD did not differ significantly between the PD and MSA-P cohorts. All subsequent analyses used the EEG functional connectivity analysis workflow summarized in Fig. 1, which generated frequency-specific AEC and wPLI connectivity matrices for group comparisons, clinical association analysis, and machine learning classification.

**Table 1:** Clinical information of patients with Parkinson's disease (PD), multiple system atrophy (MSA), and healthy controls (HC).

	HC (*n* = 27)	PD (*n* = 28)	MSA (*n* = 42)		Overall *P*
			MSA-P (*n* = 19)	MSA-C (*n* = 23)	
Age (y)	66.2 ± 7.2	66.9 ± 7.8	66.2 ± 8.2	62.8 ± 8.8	ns
Gender (M / F)	18 / 9	16 / 12	7 / 12	14 / 9	ns
Years of education	10.1 ± 2.4 (n = 23)	10.3 ± 3.4 (*n* = 25)	11.7 ± 4.2	11.7 ± 3.5 (*n* = 20)	ns
Handedness (L / R / Mixed)	1 / 25 / 0 (*n* = 26)	2 / 24 / 0 (n = 26)	1 / 16 / 0 (*n* = 17)	2 / 18/ 1 (*n* = 21)	ns
LEDD (mg / day)	–	533.8 ± 368.2 ^a^	517.5 ± 398.6 ^a^	108.7 ± 171.0	<0.001
Disease duration (y)	–	4.7 ± 2.5 ^b^	3.2 ± 2.2	2.3 ± 1.6	0.001
MDS-UPDRSIII	–	33.0 ± 13.3 (n = 26)	46.3 ± 12.7 ^b,c^ (*n* = 18)	31.4 ± 13.5	0.002
UMSARSII	–	–	21.2 ± 4.8 (n = 18)	17.5 ± 7.3	ns
NMSQ	–	15.0 ± 8.8 (*n* = 23)	19.4 ± 11.3 (*n* = 16)	15.7 ± 6.1 (*n* = 18)	ns

Note: Data are shown as mean ± standard deviation (SD). Age and gender were recorded at the time of EEG acquisition, and disease duration was calculated from the onset of motor symptoms. Overall *P* values were obtained using the Chi-square test for the gender or using the Kruskal-Wallis test for other variables. Pairwise comparisons were performed using Dunn's test with False Discovery Rate (FDR) correction. Superscripts indicate significant post hoc differences after FDR correction: ^a^*P* < 0.001 compared to MSA-C; ^b^*P* < 0.01 compared to MSA-C; ^c^*P* < 0.01 compared to PD. Clinical scores, years of education, and handedness are unavailable for some patients recruited prior to the inclusion of these specific tests in the study protocol. Abbreviations: LEDD, Levodopa equivalent daily dose; MDS-UPDRS III, Movement Disorder Society Unified Parkinson's Disease Rating Scale part III; UMSARSII, Unified Multiple System Atrophy Rating Scale Part II; NMSQ, Non-Motor Symptoms Questionnaire.

### 3.2 Distinct signatures of amplitude- and phase-based functional network disruptions in PD and MSA

We first investigated whether PD and MSA subtypes exhibit distinct source-level EEG network alterations relative to HCs. To test this, we compared whole-brain functional connectivity differences between groups using the network-based statistic (NBS) framework (Zalesky et al., 2010) combined with general linear models (GLM), controlling for age, gender, and the recording device. Across all significant contrasts, patient groups consistently exhibited functional hypoconnectivity (represented by cool colors) relative to HCs, reflecting a widespread breakdown in cortical network integration (Fig. 2). However, the nature and frequency distribution of these disruptions differed markedly between MSA and PD.

**Fig. 2:**
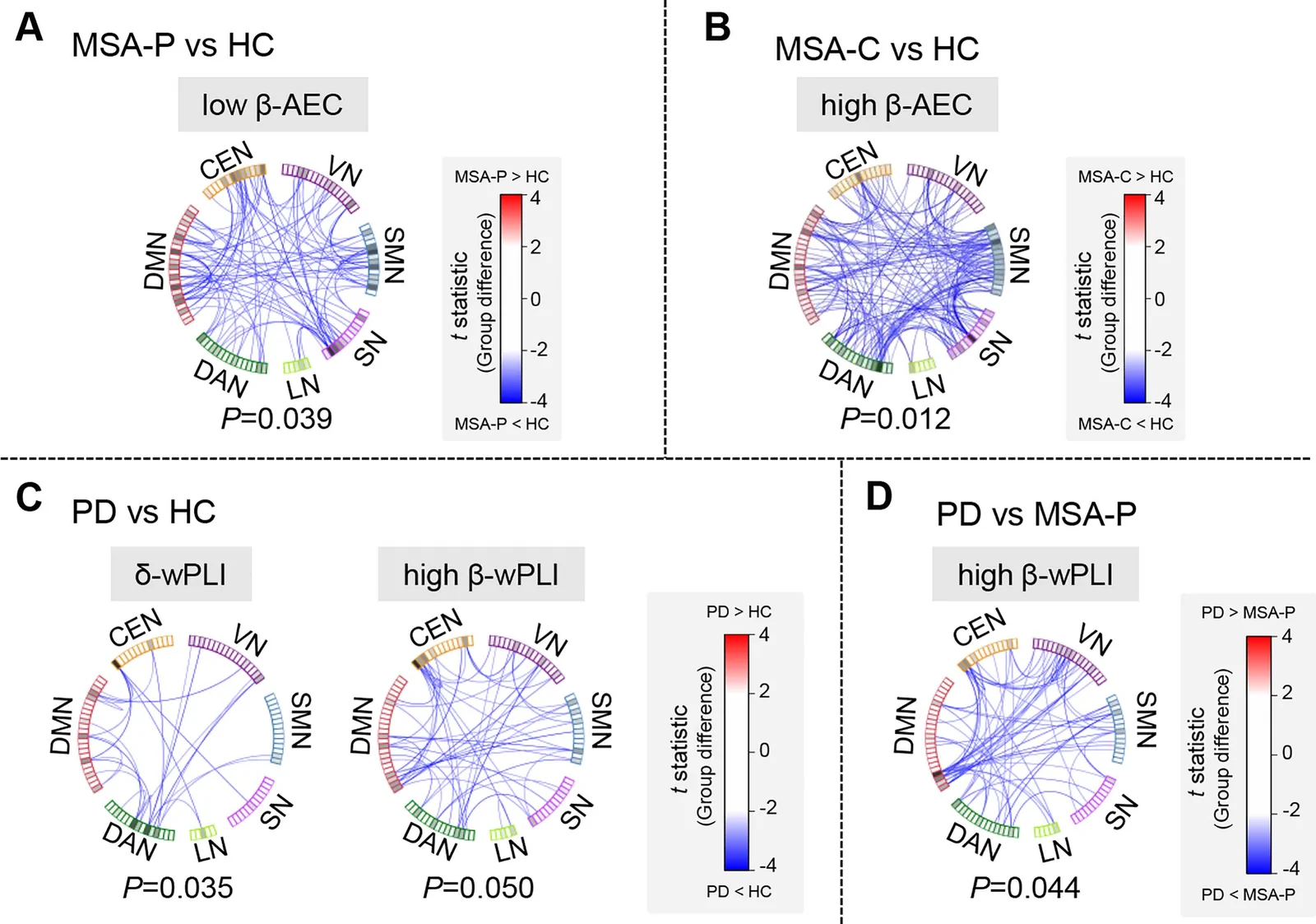
Divergent functional network alterations in PD and MSA using amplitude envelope correlation (AEC) and weighted phase-lag index (wPLI). (A–D) Group effects for functional connectivity between MSA-P and HC (A), between MSA-C and HC (B), between PD and HC (C), and between MSA-P and PD (D). The brain networks display the subnetworks identified via NBS testing for group differences. Nodes are organized into 7 canonical resting-state networks (DAN, DMN, CEN, LN, VN, SMN, SN). Edge colors indicate the *t*-statistic for the group contrast; warm colors represent higher connectivity in the first group compared to the second, while cool colors represent lower connectivity. Statistical significance was determined using the NBS procedure, with edge-level *t*-statistics derived from general linear models controlling for nuisance covariates (age, gender, and recording device).

In patients with MSA, significant network disruptions were characterized by reduced amplitude-based coupling within the β frequency ranges. Specifically, the MSA-P subtype exhibited a significant subnetwork of hypoconnectivity in the low β band (FWER-corrected *P* = 0.039; Fig. 2A), primarily disrupting inter-network communication among the DMN, CEN, SMN, and SN. The MSA-C subtype demonstrated marked amplitude-based hypoconnectivity in the high β band (*P* = 0.012; Fig. 2B), heavily disrupting the DMN, CEN, SMN, SN, and DAN. However, a direct NBS contrast between the MSA-P and MSA-C groups yielded no significant differences in either the low-β or high-β band, providing no evidence of a formal frequency-specific dissociation between the two MSA subtypes. Neither MSA subtype showed significant phase-based network alterations relative to HCs.

For patients with PD, a pattern of functional hypoconnectivity involving phase coupling compared to HCs was observed (Fig. 2C). Compared to HCs, the PD group demonstrated phase-based decoupling (wPLI) in the δ (*P* = 0.035) and the high β bands (*P* = 0.050), mainly affecting the DMN, CEN, DAN, VN, and SMN. Because MSA-P shares highly overlapping clinical presentations with PD, making differential diagnosis difficult, we subsequently evaluated the direct network differences between these two specific cohorts. PD exhibited lower wPLI connectivity within a high β-band subnetwork than MSA-P (*P* = 0.044, Fig. 2D). Patient-only sensitivity analyses revealed that this high β-band phase decoupling in PD remained consistent when additionally adjusting for LEDD (*P* = 0.043) and became more topographically dense when additionally adjusting for disease duration and MDS-UPDRS III (*P* = 0.028, Supplementary Fig. S2). These results indicate that the observed network differences between PD and MSA-P reflect intrinsic, disease-specific pathophysiological differences rather than artifacts of medication dosage, disease stage, or symptom severity. Together, functional connectivity analysis with NBS identified distinct frequency- and topology-specific network disruptions in PD and MSA subtypes.

### 3.3 Shared functional network correlates of motor and non-motor symptoms

Because PD and MSA share overlapping motor and non-motor symptoms, we next asked whether symptom severity maps onto common functional network alterations across diagnoses. We evaluated the main effect of clinical scores using the established GLM framework, with the addition of diagnosis as a covariate to isolate the continuous effect of symptom severity from baseline group differences. Correlation analysis of the MDS-UPDRS III subdomain scores revealed that bradykinesia and tremor are associated with distinct, frequency-specific network alterations (Fig. 3). Greater bradykinesia severity was strongly associated with widespread phase-based decoupling in an α-band subnetwork (FWER-corrected *P* = 0.003; Fig. 3A). This negative correlation followed a consistent, parallel associations in both PD (*r*_*p*_ = −0.440) and MSA (*r*_*p*_ = −0.591). Conversely, higher tremor scores correlated with amplitude-based hypoconnectivity in the low β band (*P* = 0.012, Fig. 3B).

**Fig. 3:**
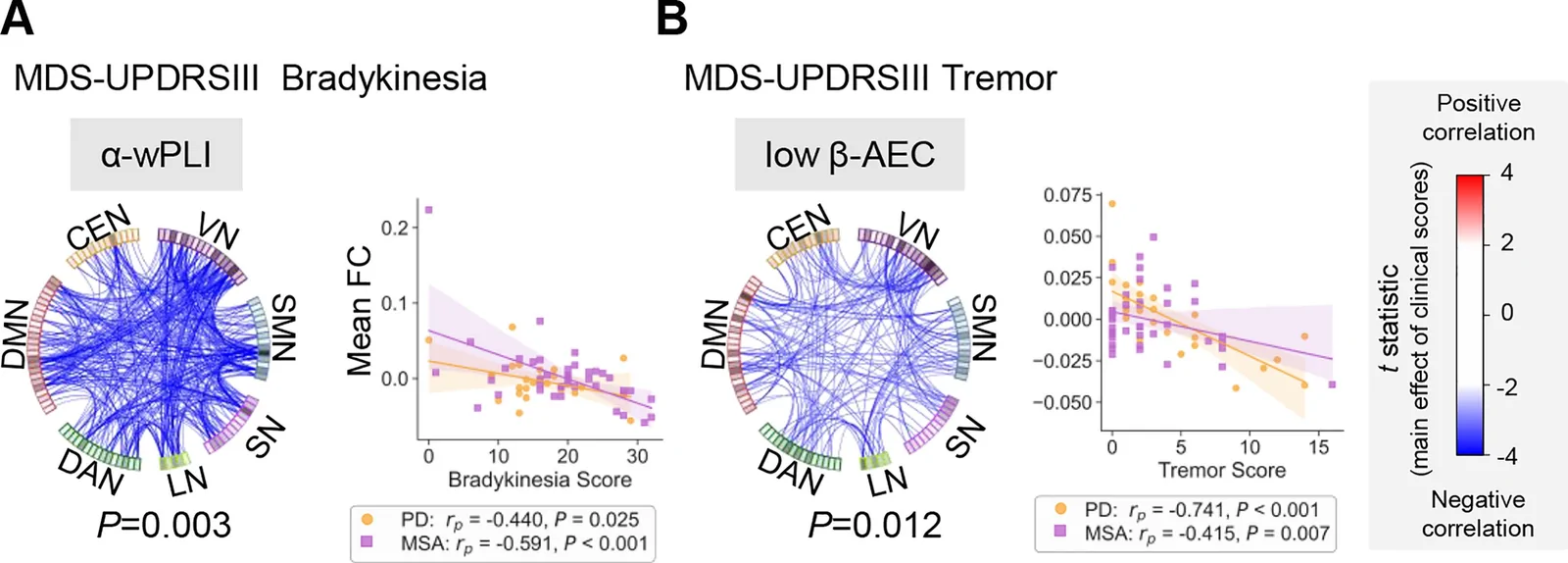
Shared functional network correlates of motor symptoms in PD and MSA. (A–B) Main effects of clinical scores on functional connectivity for MDS-UPDRS III sub-scores: bradykinesia (A) and tremor (B). For each panel, brain networks (top) display the subnetworks identified via NBS testing for the shared association between symptom severity and connectivity across both disease groups. Nodes are organized into 7 canonical resting-state networks (DAN, DMN, CEN, LN, VN, SMN, SN). Edge colors indicate the main effect *t*-statistic for the clinical score: warm colors denote positive correlations (increased connectivity with greater symptom severity), whereas cool colors denote negative correlations. Scatter plots (bottom) illustrate the relationship between the extracted mean subnetwork connectivity and the corresponding clinical score for the PD and MSA groups. To isolate the effect of symptom severity from baseline disease differences, statistical significance of the subnetworks was determined using general linear models controlling for diagnosis and nuisance covariates (age, gender, and recording device). Plotted functional connectivity values on the y-axis represent adjusted residuals from these models. Partial correlation coefficients (*r*_*p*_) are provided descriptively to visualize the consistent, parallel clinical associations within each specific diagnostic group. Note that for all clinical assessments, higher scores indicate greater symptom severity.

Beyond motor impairments, we assessed the shared network correlates of non-motor symptoms measured by the NMSQ (Supplementary Fig. S3). Interestingly, while motor symptoms were primarily linked to functional decoupling, the deterioration of non-motor symptoms was predominantly characterized by functional hyperconnectivity within slow-wave (δ, θ). Specifically, increased autonomic burden was significantly associated with elevated amplitude-based coupling in the θ band (*P* = 0.013; Supplementary Fig. S3A), while higher psychiatric and cognitive symptom scores correlated with elevated phase-based and amplitude-based coupling in the δ band, respectively (*P* = 0.027 for the δ-band wPLI subnetwork and 0.020 for the δ-band AEC subnetwork; Supplementary Fig. S3B, C). Across all non-motor domains, the scatter plots demonstrated shared trends between the PD and MSA cohorts. These findings reveal symptom-associated network signatures that are detectable across both disorders, suggesting shared functional network components of motor and non-motor impairment in PD and MSA.

### 3.4 Divergent functional network correlates of symptom severity in PD and MSA

While the previous analyses identified shared neural correlates, the distinct clinical progression and treatment responsiveness of PD and MSA suggest they likely also possess divergent pathophysiological trajectories. To further investigate disease-specific brain-behavior relationships, we evaluated the group × clinical score interaction effect using NBS, allowing us to identify subnetworks where the correlation between symptom severity and functional connectivity significantly differed between the two disorders.

Analysis of motor impairments revealed bidirectional dissociations in the relationship between functional networks and symptom severity (Fig. 4). For bradykinesia, we identified a significant interaction in θ-band phase coupling (FWER-corrected *P* = 0.030; Fig. 4A), indicating opposed relationships where worsening bradykinesia was strongly associated with increased θ-band synchronization in PD (*r*_*p*_ = 0.585) but decreased synchronization in MSA (*r*_*p*_ = −0.483). Conversely, resting tremor exhibited the opposite interaction pattern. Tremor severity showed a significant interaction in a dense amplitude-based subnetwork in the high β-band (*P* = 0.012; Fig. 4B), where worsening tremor correlated with decreased connectivity in PD (*r*_*p*_ = −0.525) but increased connectivity in MSA (*r*_*p*_ = 0.458).

**Fig. 4:**
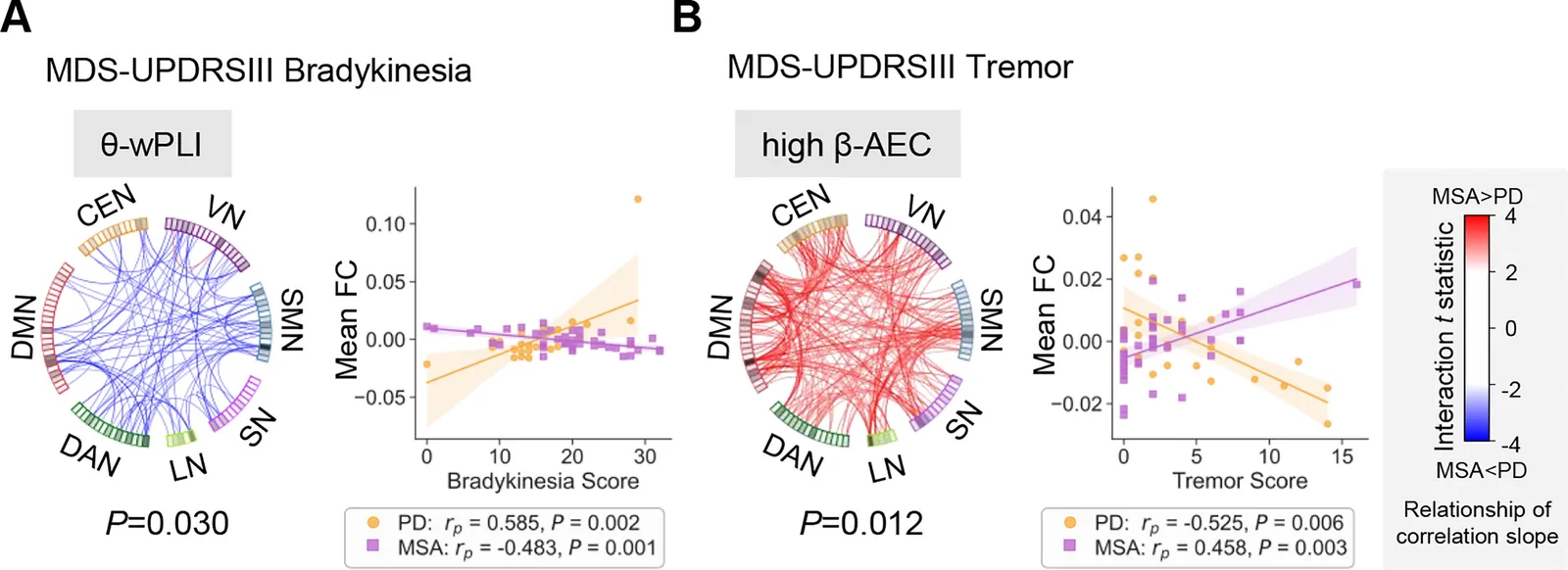
Divergent functional network correlates of motor symptoms in PD and MSA. (A–B) Group × Score interaction effects for functional connectivity and MDS-UPDRS III sub-scores: bradykinesia (A) and tremor (B). For each panel, brain networks (top) display the subnetworks identified via NBS interaction testing. Edge colors indicate *t*-statistics for the interaction term: warm colors denote connections where the association between symptom severity and connectivity is significantly more positive in MSA compared to PD, whereas cool colors denote a more positive association in PD. Statistical significance was determined using the NBS procedure, with edge-level interaction *t*-statistics derived from general linear models controlling for nuisance covariates (age, gender, and recording device). Scatter plots (bottom) illustrate the relationship between the extracted mean subnetwork connectivity and the corresponding clinical score for the PD and MSA groups. Plotted mean FC values are residuals adjusted for nuisance covariates. While NBS provided the primary inferential framework for the interaction effect, partial correlation coefficients (*r*_*p*_) are provided descriptively to visualize the group-specific clinical associations. Note that for all clinical assessments, higher scores indicate greater symptom severity.

Regarding non-motor symptoms, we observed a significant divergent association for cognitive deterioration (Supplementary Fig. S4). Worsening cognitive symptom burden on the NMSQ was associated with a significant group × score interaction in a θ-band phase coupling subnetwork (*P* = 0.020), where connectivity increased with symptom severity in PD (*r*_*p*_ = 0.639) but decreased in MSA (*r*_*p*_ = −0.587). Together, these interaction analyses indicate that, beyond the shared symptom-associated signatures identified above, PD and MSA exhibit divergent relationships between functional connectivity and symptom severity.

### 3.5 Machine-learning classification using EEG network features

Previous group-level analyses revealed distinct EEG functional network alterations between PD and MSA, suggesting that source-level EEG connectivity may contain disease-specific electrophysiological information. Given the major clinical challenge of distinguishing MSA-P from PD, we next explored whether EEG functional network features provide complementary diagnostic value beyond standard clinical assessments. Pursuing this, we employed a target residual learning framework (Supplementary Fig. S5). Rather than evaluating EEG and clinical features in isolation, this approach specifically tests whether EEG can correct the predictive errors made by a baseline clinical model.

The base model, trained on clinical features (disease duration, modified Hoehn & Yahr scale, and MDS-UPDRS III sub-scores) alone, achieved a moderate diagnostic performance with an overall accuracy of 76.59 ± 16.70%, precision of 73.33 ± 29.04%, sensitivity of 71.42 ± 30.16%, and specificity of 80.13 ± 22.53% (Table 2). Adding EEG features modestly improved overall performance across repeated cross-validation, yielding an accuracy of 79.08 ± 15.97%, precision of 76.23 ± 29.05%, and specificity of 84.28 ± 19.49%. Sensitivity remained comparable between the two models. To determine whether these gains reflected meaningful EEG information rather than the effect of adding high-dimensional features, we evaluated the results against a null distribution generated by randomly shuffling the EEG features. The performance gain made by adding true EEG features outperformed adding shuffled EEG features for accuracy (permutation test*, P* = 0.039), precision (*P* = 0.049), and sensitivity (*P* = 0.048). However, it is important to note that the absolute incremental gains were small (e.g., 2.50% for accuracy and 0.13% for sensitivity). The permutation significance suggests that true EEG features contribute some complementary information, though this does not yet establish a clinically meaningful improvement in diagnostic performance.

**Table 2:** Performance of the machine learning classifiers for differentiating PD and MSA-P.

	Accuracy	Precision	Sensitivity	Specificity	F1 score
**Clinical Features** (mean ± SD_CV_, %)	76.59 ± 16.71	73.33 ± 29.04	71.42 ± 30.16	80.12 ± 22.53	69.00 ± 25.04
**Clinical + EEG Features** (mean ± SD_CV_, %)	79.08 ± 15.97	76.23 ± 29.05	71.54 ± 30.40	84.28 ± 19.49	70.81 ± 25.77
**Observed Δ** (%)	2.50	2.90	0.13	4.16	1.81
**Shuffled-EEG null Δ** (mean ± SD_perm_, %)	−1.88 ± 2.35	−3.31 ± 3.63	−8.04 ± 4.33	2.45 ± 2.28	−5.61 ± 3.81
**Permutation *P* value**	0.039	0.049	0.048	0.228	0.038

Note: The combined model trained on both clinical and EEG features is compared to the base model trained on clinical features alone. Performance metrics are evaluated using 100-repeat stratified 8-fold cross-validation and shown as mean ± SD_CV_ across all the 800 test folds. Sensitivity and specificity are reported with respect to MSA-P as the positive class. Observed Δ denotes the mean performance gain of the combined model. Shuffled-EEG null Δ denotes the null distribution of the mean performance gain, which is generated by randomly shuffling the EEG features and rerun the full cross validation pipeline for 1000 permutations, presented as mean ± SD_perm_ across all the permutations. The Permutation *P* value represents the proportion of permutations in which the model trained on shuffled EEG features achieved a mean performance gain equal to or greater than the model trained on true EEG features.

To characterize how the features influence the model's predictions, we evaluated feature contributions using SHAP values (Lundberg and Lee, 2017) (Fig. 5). The model's primary drivers aligned well with established clinical observations: shorter disease duration, higher modified Hoehn & Yahr scale, and severe midline/bradykinetic symptoms drove predictions toward MSA-P, whereas prominent tremor strongly shifted predictions toward PD. Importantly, several EEG features ranked among the features with the largest mean absolute SHAP values, indicating that these features contributed to the predictions within the fitted models. Specifically, elevated phase-based coupling in the high β band (among SMN, VN, DMN, LN, and DAN) and δ band (between SN and CEN), alongside θ-band amplitude-based coupling within LN, emerged as key differentiators pushing predictions toward MSA-P. The prominent ranking of these EEG functional network features underscores their potential as objective biomarkers for resolving this specific diagnostic overlap.

**Fig. 5:**
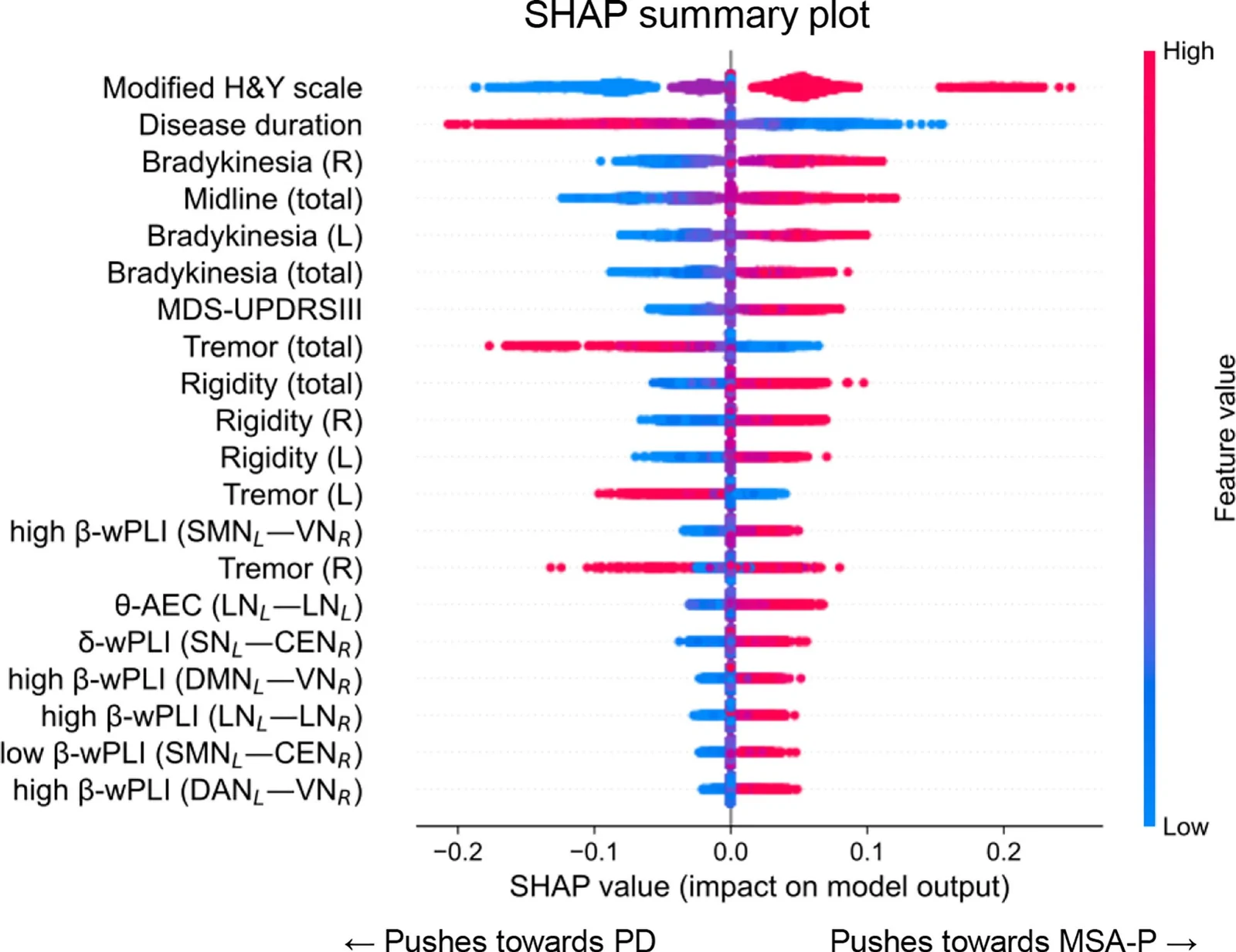
Feature importance scores for the machine learning models trained to discriminate between PD and MSA-P. The plot displays the top 20 most discriminative features (including clinical and EEG functional connectivity features), ranked by their mean absolute SHAP values across 800 cross-validation folds. Each dot represents a single test sample prediction (*n* = 4400 total points, representing repeated hold-out predictions of the 44 participants across the 100 cross-validation repeats). The position on the horizontal axis indicates the SHAP value. Positive values drive the model's prediction toward MSA-P, while negative values drive the prediction toward PD. The color gradient reflects the normalized value of the original feature for that specific sample, with red indicating a higher value (e.g., higher clinical score or stronger connectivity) and blue indicating a lower value.

## 4 Discussion

In this study, we used source-reconstructed resting-state EEG to characterize frequency-specific functional network alterations in PD and MSA. By integrating a data-driven masking EMD approach with leakage-corrected connectivity metrics, we identified frequency- and metric-specific functional network disruptions that effectively distinguish PD from MSA subtypes. We demonstrated that while both disorders shared common network correlates for specific clinical impairments (e.g., bradykinesia and non-motor decline), they also exhibited divergent network associations with symptom severity, likely reflecting the distinct capacities for cortical reorganization of PD and MSA. Finally, using a target residual machine-learning framework, we provided a proof-of-concept demonstration that EEG network features capture subclinical pathophysiological variance, improving the performance of differentiating MSA-P from PD over clinical assessments alone. Together, these findings highlight the potential of source-level EEG function network features as candidate biomarkers for atypical parkinsonism.

### 4.1 Divergent patterns of amplitude and phase network disruptions

Our NBS analysis identified widespread functional hypoconnectivity in both PD and MSA compared to HCs; however, the patterns of these disruptions exhibited different frequency and metric profiles in separate patient-control comparisons. MSA, encompassing both MSA-P and MSA-C subtypes, demonstrated amplitude decoupling within the β band. In contrast, PD exhibited a broader pattern of phase decoupling across the δ and β bands.

Amplitude- and phase-based connectivity quantify related but distinct aspects of large-scale oscillatory coordination (Siems and Siegel, 2020). AEC captures slow, modulatory co-activations of brain regions, which are heavily dependent on the large-scale white matter integrity (Cabral et al., 2014; Messaritaki et al., 2021). MSA is primarily an oligodendrogliopathy characterized by widespread glial cytoplasmic alpha-synuclein inclusions, leading to prominent demyelination and structural white matter degeneration across striatonigral and olivopontocerebellar tracts (Ogawa et al., 2021; Poewe et al., 2022). This profound structural damage may directly impair the broad, envelope-based modulatory communication across the cortex. However, from the perspective of oscillatory dynamics, reduced envelope correlation does not strictly require anatomical disconnection; it may also arise from an altered temporal organization of oscillatory bursts or a reduced signal-to-noise ratio in local circuits (Seedat et al., 2020). Less frequent or less consistently coordinated β bursts could reduce envelope covariation even if the underlying structural pathways remain partially intact.

Furthermore, while separate comparisons with HCs showed β-band amplitude disruptions predominantly in the low β (<20 Hz) range for MSA-P and high β (>20 Hz) for MSA-C, these findings describe distinct subgroup patterns rather than a formally tested group×frequency interaction. Nevertheless, this differentiated detectability of hypoconnectivity in MSA-P and MSA-C intriguingly parallels their distinct topographies of neurodegeneration. Given that recent neurophysiological evidence suggested low and high β oscillations possess distinct network origins and functional roles (Cao et al., 2024; Nougaret et al., 2024), these observed patterns may reflect the distinction between the predominant striatonigral pathology of MSA-P and the profound olivopontocerebellar involvement in MSA-C.

In contrast, PD demonstrated additional phase-based decoupling within the δ and β bands, which is in line with previous literature (Conti et al., 2022; Yassine et al., 2022; Zhang et al., 2022). wPLI quantifies the consistency of non-zero-lag phase relationships. Dynamically, a reduction in wPLI may reflect greater variability in relative phase timing, increased frequency mismatch between regions, or changes in the intermittency of coordinated oscillations. The focal loss of dopaminergic neurons in the substantia nigra in PD may disrupt the precise phase-timing and entrainment of the basal ganglia–thalamocortical circuits, resulting in more variable and less consistent cortical synchronization (Wang et al., 2021).

### 4.2 Shared clinical correlates of motor and non-motor symptoms in PD and MSA

Despite their distinct primary pathologies, PD and MSA share several overlapping clinical phenotypes. Our analysis of shared clinical correlates revealed that bradykinesia was associated with widespread phase-based decoupling in the α-band. Cortical α-rhythms are fundamental for sensorimotor integration and the top-down gating of motor networks (Sadaghiani et al., 2012; Rhodes et al., 2018), and higher α-band synchronization can facilitate cortical signal transmission (Zazio et al., 2021). In the context of parkinsonism, reduced α-band coupling may therefore indicate impaired integration among motor, executive, attentional, and default-mode systems needed for efficient movement initiation and control, suggesting that loss of α-band synchronization is a shared indicator of motor execution failure across PD and MSA. In addition, tremor showed a different network signature from bradykinesia. Greater tremor severity was associated with reduced low β amplitude. Tremor in parkinsonian disorders is thought to involve both the basal ganglia and the cerebello–thalamo–cortical circuit (Helmich et al., 2012; Zhong et al., 2022). In this context, the amplitude decoupling in the low β band may reflect disrupted motor-network modulation (Qasim et al., 2016) that triggers the tremor-related pathological synchronization (Hirschmann et al., 2013).

While motor symptoms correlated with network decoupling, the deterioration of non-motor symptoms (autonomic, cognitive, and psychiatric) was predominantly linked to functional hyperconnectivity in the slow-wave (δ and θ) bands. Although this symptom-related hyperconnectivity might seem to contrast with the baseline δ-band hypoconnectivity observed in PD patients relative to healthy controls, it may reflect a transition from the loss of healthy network integration to the emergence of pathological hypersynchronization. Previous studies have linked localized slow-wave abnormalities to cognitive decline in PD and other neurodegenerative conditions (Benz et al., 2014; Pal et al., 2020; Soikkeli et al., 1991). Our results extend these findings to large-scale network dynamics. We speculate that as non-motor symptoms worsen, widespread cholinergic denervation, which is a hallmark of cognitive and psychiatric decline in alpha-synucleinopathies, might disrupt normal cortical desynchronization (Gratwicke et al., 2015), forcing the neural populations into rigid, hyper-synchronous slow-wave coupling. Therefore, rather than a compensatory return to healthy function, this slow-wave hyperconnectivity likely represents a malfunctioning, locked state that restricts the brain's normal adaptive dynamics, manifesting as profound cognitive and psychiatric impairment.

### 4.3 Diagnosis-dependent associations between connectivity and symptom burden

An interesting finding of our study is the identification of significant group × clinical score interactions, which revealed divergent brain-behavior relationships between PD and MSA. For instance, worsening bradykinesia and cognitive impairments were strongly associated with increased θ-band synchronization in PD, but decreased synchronization in MSA. Similarly, worsening tremor correlated with decreased high-β AEC in PD, but increased connectivity in MSA.

These bidirectional associations provide insights into how network dysfunction manifests differently across these disorders. In PD, the observed hyper-synchronization in the θ-band associated with greater symptom severity could hypothetically represent an adaptive attempt to recruit auxiliary cortical networks to overcome basal ganglia deficits and maintain motor and cognitive output (Blesa et al., 2017; Palmer et al., 2010). However, a cross-sectional positive association with symptom severity does not inherently demonstrate compensation. Alternatively, this hyper-connectivity may reflect maladaptive pathological synchronization, such as the loss of healthy cortical desynchronization, or a breakdown in network flexibility. In contrast, the widespread, rapidly progressive neurodegeneration in MSA may preclude these synchronous states, leading to the observed decreases in connectivity among patients with greater symptom burden. These divergent associations underscore the necessity of moving beyond cross-sectional group differences to understand disease-specific brain-behavior relationships. Ultimately, distinguishing whether these cross-sectional network associations reflect compensatory adaptations or purely maladaptive decline will require longitudinal tracking to evaluate whether such hyper-connectivity actually preserves clinical function over time.

### 4.4 Exploratory classification and incremental discriminative value of EEG functional networks

Clinically, differentiating MSA-P from PD remains challenging due to overlapping parkinsonian features. The exploratory machine learning analysis provides preliminary evidence that EEG functional connectivity may contain diagnostic information not fully captured by standard clinical variables. The target residual learning framework, inspired by the idea of gradient boosting (Friedman, 2001), was intentionally designed to test whether EEG features could correct the residual errors of a clinical model rather than simply compete with clinical assessment. The addition of EEG features yielded a modest improvement in overall accuracy, precision, and specificity for distinguishing MSA-P from PD in the internal validation study, providing a proof-of-concept that resting-state EEG connectivity may offer incremental, complementary value for differential diagnosis.

Importantly, these classification results must be interpreted with caution. The classifier utilized a relatively small sample (47 participants) relative to the high-dimensional EEG feature space. While we employed rigorous cross-validation for a stable estimate of internal performance and used regularization to prevent overfitting, the incremental clinical gains observed were small. Furthermore, while the permutation analysis confirmed that true EEG features outperformed randomly shuffled EEG features, this demonstrates that EEG features contributed complementary information in our classification model rather than establishing a significant improvement in clinical diagnostic utility. The interpretability provided by SHAP values confirmed that the model's predictions were driven by biologically plausible features, aligning closely with our group-level network findings (e.g., high β- and δ-band connectivity). However, this proof-of-concept internal validation requires testing in larger, independent, multi-center cohorts before any true clinical diagnostic application can be considered.

### 4.5 Methodological strengths and limitations

Several methodological features strengthen the present study. We analyzed EEG in source space and organized cortical signals according to canonical functional networks, allowing interpretation at a systems-neuroscience level rather than at the sensor level alone. In addition, standard Fourier or wavelet-based methods often struggle with the non-stationary nature of resting-state EEG. By employing Masking EMD (Huang et al., 1998; Deering and Kaiser, 2005), we effectively mitigated mode-mixing, isolating canonical frequency bands in a data-driven yet cross-subject and cross-ROI aligned manner. Furthermore, the strict application of leakage-corrected connectivity metrics, orthogonalized AEC (Hipp et al., 2012) and wPLI (Vinck et al., 2011), ensures that our findings should represent true large-scale neural interactions instead of spurious volume conduction artifacts. Additionally, by evaluating patients in the off-medication state, and further confirming the topological stability of our results of group differences through LEDD-adjusted sensitivity analyses, we minimized the risk of transient medication-related confounding.

Nevertheless, several limitations must be acknowledged, necessitating a cautious, proof-of-concept interpretation of the results. First, the overall sample size is relatively modest, particularly for MSA, which is much rarer than PD. As discussed above, this particularly constrains the machine learning classification, which must be viewed strictly as an exploratory internal validation. Larger, independent, multi-center cohorts are required to establish the generalizability of both the group-level network differences and the predictive models. Second, due to the cross-sectional design of the study, whether the shared and divergent clinical associations represent the true evolving trajectories remains unclear. Longitudinal EEG tracking is required to definitively map these dynamic network alterations over time. Third, the individual NBS analysis did not control for family-wise error across multiple frequency bands, connectivity metrics, group contrasts, and clinical scales. Therefore, the reported findings map the topological distribution of effects within an exploratory framework, and independent validation in larger cohorts is required to confirm these specific network signatures. Fourth, while acute medication effects were controlled for, the residual neuroplastic effects of chronic dopaminergic therapy cannot be entirely excluded. However, because a levodopa trial is clinically required to establish the differential diagnosis of MSA, chronic medication exposure remains an inherent and unavoidable confounding factor in comparative studies of parkinsonian disorders. Finally, while eLORETA combined with leakage correction is highly effective, the spatial resolution of 64-channel EEG and the use of a template MRI instead of individual MRIs inherently limit the anatomical precision of the cortical parcellation.

## 5 Conclusion

In conclusion, this exploratory study demonstrates that source-level EEG functional connectivity can reveal distinct, frequency-specific network disruptions in PD and MSA subtypes. By distinguishing between amplitude- and phase-based coupling, and by identifying both shared and divergent clinical associations, we provide new insights into the distinct patterns of network dysfunction underlying PD and MSA. Combined with machine learning, these electrophysiological fingerprints offer objective and non-invasive imaging biomarkers that hold potential for improving the differential diagnosis and management of atypical parkinsonian disorders. Replication and prospective validation are the essential next steps toward clinical translation.

## Data and code availability statement

EEG data are not publicly available due to ethical restrictions. The derived EEG features and the code used to produce the results can be downloaded from https://github.com/chendaichao/EEG-functional-network-in-PD-and-MSA.

## Declaration of generative AI and AI-assisted technologies in the manuscript preparation process

During the preparation of this work the author(s) used Gemini 3.1 pro (Google) and GPT 5.5 (OpenAI) in order to check the grammar and improving the clarity of the text. After using this tool/service, the author(s) reviewed and edited the content as needed and take(s) full responsibility for the content of the published article.

## CRediT authorship contribution statement

**Daichao Chen:** Writing – review & editing, Writing – original draft, Visualization, Software, Methodology, Investigation, Formal analysis, Data curation, Conceptualization. **Huada Tang:** Methodology, Investigation, Data curation, Conceptualization. **Yanfei Pan:** Investigation. **Zhuangzhuang Zhang:** Investigation. **Wenzhe Wang:** Investigation. **Fangda Leng:** Methodology, Conceptualization. **Haotian Yan:** Methodology, Conceptualization. **Hui Sang:** Investigation. **Kai Li:** Resources, Data curation. **Fan Li:** Resources, Data curation. **Yunchuang Sun:** Resources, Data curation. **Jing Chen:** Resources, Data curation. **Wei Sun:** Resources, Data curation. **Jue Zhang:** Supervision, Methodology. **Zhaoxia Wang:** Writing – review & editing, Supervision, Resources, Project administration, Funding acquisition, Conceptualization. **Luhua Wei:** Writing – review & editing, Supervision, Resources, Project administration, Funding acquisition, Data curation, Conceptualization.
