# Supplementary material for: Distinct EEG functional network alterations in Parkinson's disease and multiple system atrophy
Source: Neuroimage Clin. 2026 Sep 27;52:104072. doi: 10.1016/j.nicl.2026.104072 (PMC13651690; doi:10.1016/j.nicl.2026.104072)
Supplement: Supplementary file 1 [file mmc1.pdf]

Supplementary Material

Distinct EEG functional network alterations in Parkinson's disease and multiple system atrophy

Daichao Chen [a,b], Huada Tang [a], Yanfei Pan [a], Zhuangzhuang Zhang [a], Wenzhe Wang [a], Fangda Leng [c], Haotian Yan [d], Hui Sang [e], Kai Li [a], Fan Li [a], Yunchuang Sun [a], Jing Chen [a], Wei Sun [a], Jue Zhang [b,f], Zhaoxia Wang [a,b,*], Luhua Wei [a,*]

Author affiliations:
[a] Department of Neurology, Peking University First Hospital, Beijing 100034, China
[b] Academy for Advanced Interdisciplinary Studies, Peking University, Beijing 100871, China
[c] Fein Memory and Aging Center, Department of Neurology, University of California, San Francisco, CA 94158, USA
[d] Department of Neurology, Xuanwu Hospital, Capital Medical University, Beijing 100053, China.
[e] Department of Neurology, Taiyuan City Central Hospital, Taiyuan 030009, Shanxi, China
[f] School of Mechanics and Engineering Science, Peking University, Beijing 100871, China
[*] Corresponding author at: Department of Neurology, Peking University First Hospital, Beijing 100034, China.
E-mail address: weiluhua2008@outlook.com (L. Wei), drwangzx@163.com (Z. Wang).

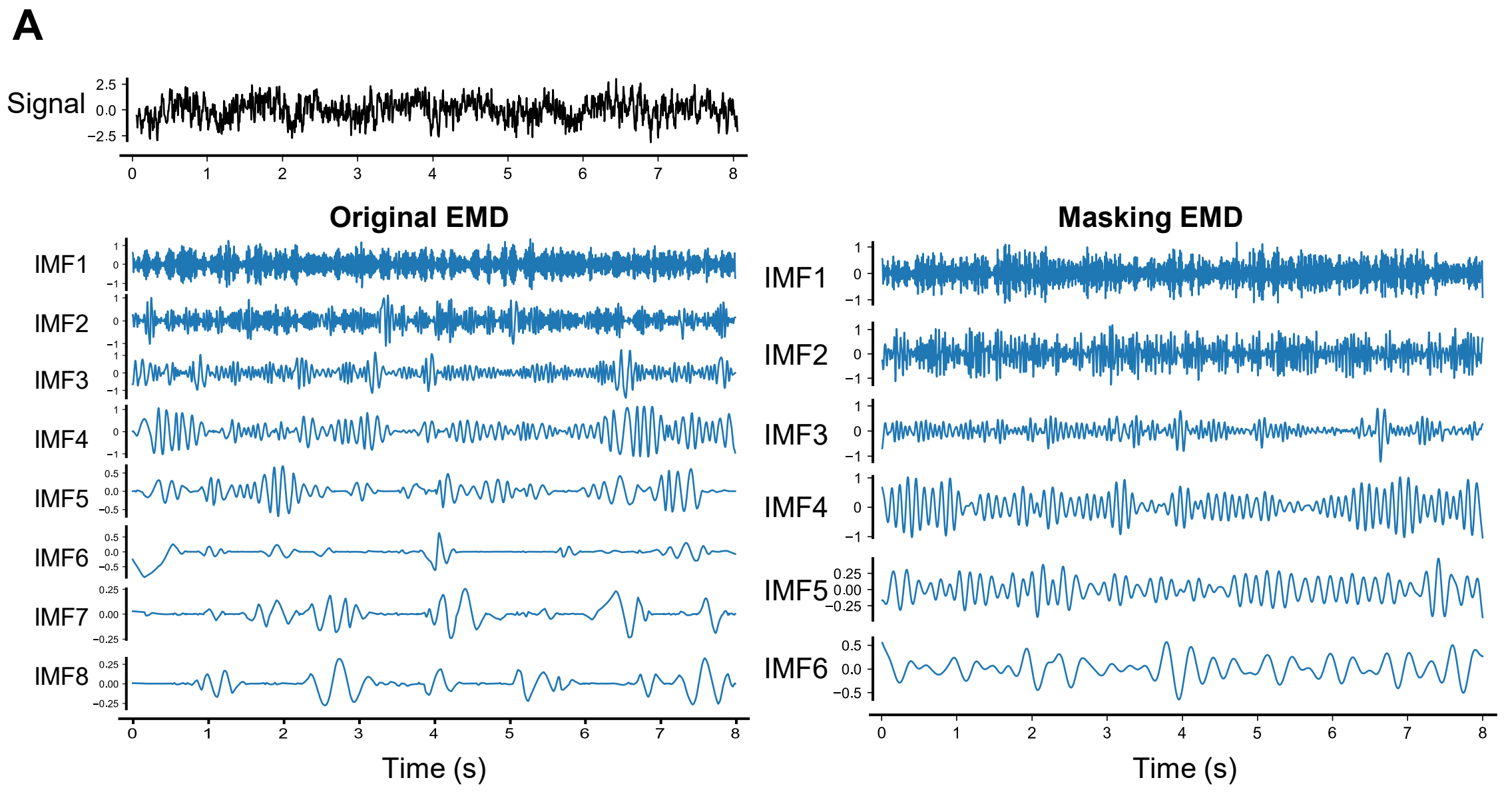

B

	IMF center frequency (Hz)	Oscillation type
IMF1	81.40 ± 1.39	discarded
IMF2	30.55 ± 1.76	→ high β
IMF3	15.04 ± 0.74	→ low β
IMF4	9.41 ± 0.29	→ α
IMF5	5.85 ± 0.10	→ θ
IMF6	3.16 ± 0.05	→ δ

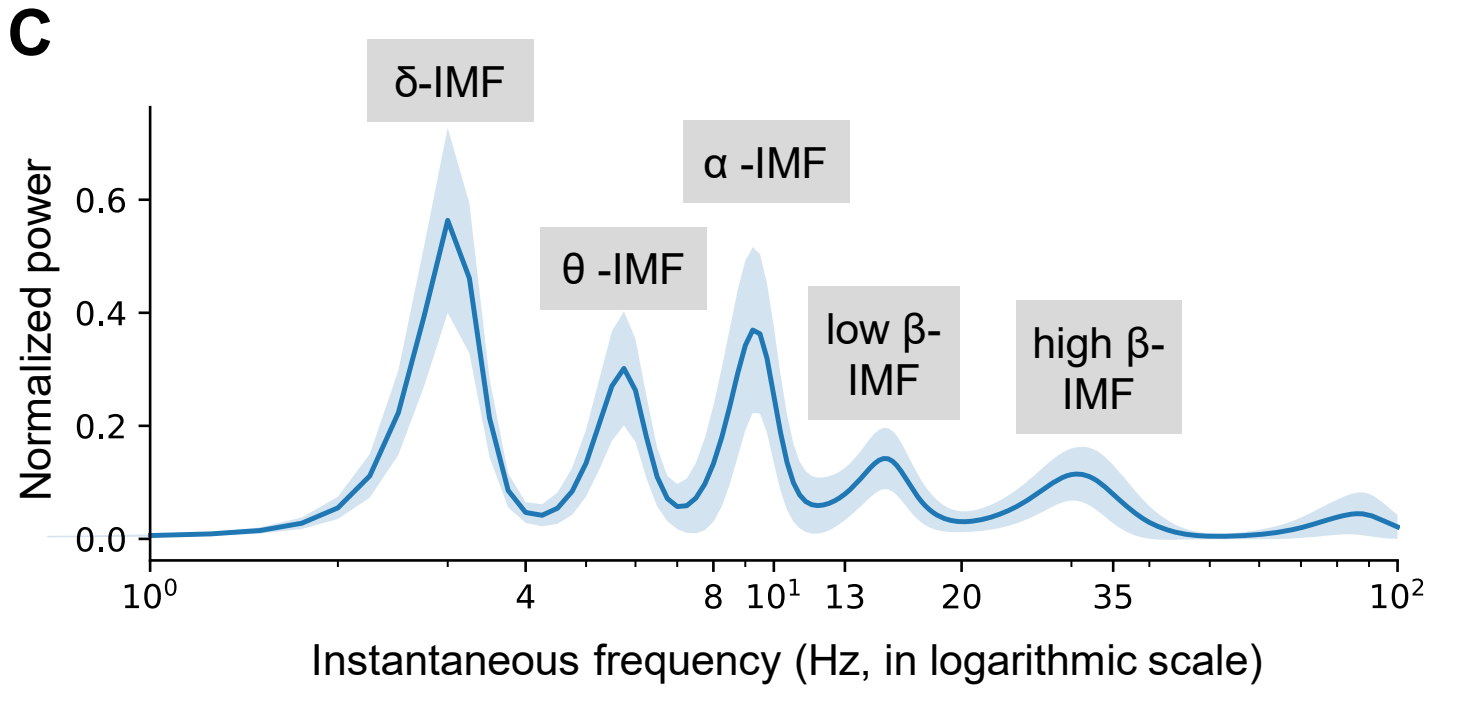

Supplementary Fig. S1 Empirical mode decomposition (EMD) of source-reconstructed EEG signals. (A) Comparison of Masking EMD and the original EMD to a representative time series from a single cortical region of interest (ROI), yielding multiple intrinsic mode functions (IMFs) from high to low frequencies. **(B)** Mapping of data-driven IMFs from Masking EMD to canonical EEG frequency bands. The center frequency of each IMF was calculated across all 100 cortical ROIs for all 97 participants and is presented as mean ± SD (n=9,700). IMF1 was discarded to exclude potential high-frequency noise and muscle artifacts. The center frequencies of IMFs 2 through 6 align closely with classical frequency bands: high β (20–35Hz), low β (13–20Hz), α (8–13Hz), θ (4–8Hz), and δ (1–4Hz), respectively. **(C)** The normalized Hilbert spectrum averaged across all ROIs and participants (n=9,700). Shaded areas represent the SD, illustrating the distinct distribution of instantaneous frequencies for the derived IMFs.

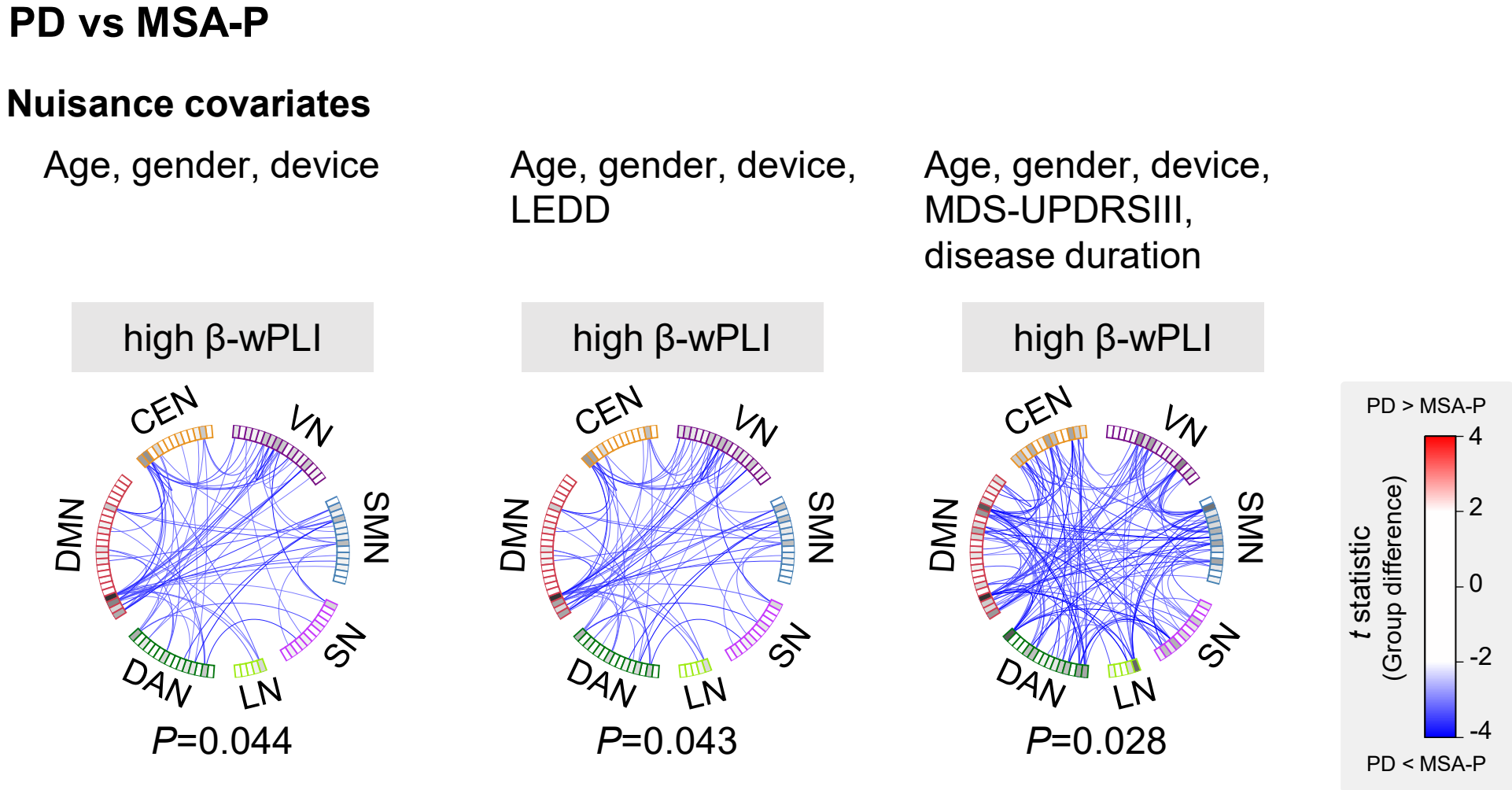

Supplementary Fig. S2 Sensitivity analyses evaluating the robustness of the functional network differences between PD and MSA-P. The figure displays the significant subnetwork of high β-band wPLI in PD compared to MSA-P, evaluated under three different general linear models to account for potential confounders. Left: The primary base model adjusting for age, gender, and recording device. Middle: A sensitivity model additionally adjusting for medication exposure using the levodopa-equivalent daily dose (LEDD). Right: A sensitivity model adjusting for clinical severity (MDS-UPDRS III) and disease duration. Edge colors indicate the *t*-statistic for the group contrast; cool colors represent lower connectivity in PD compared to MSA-P. Statistical significance was determined using the network-based statistic (NBS) procedure with 5,000 permutations.

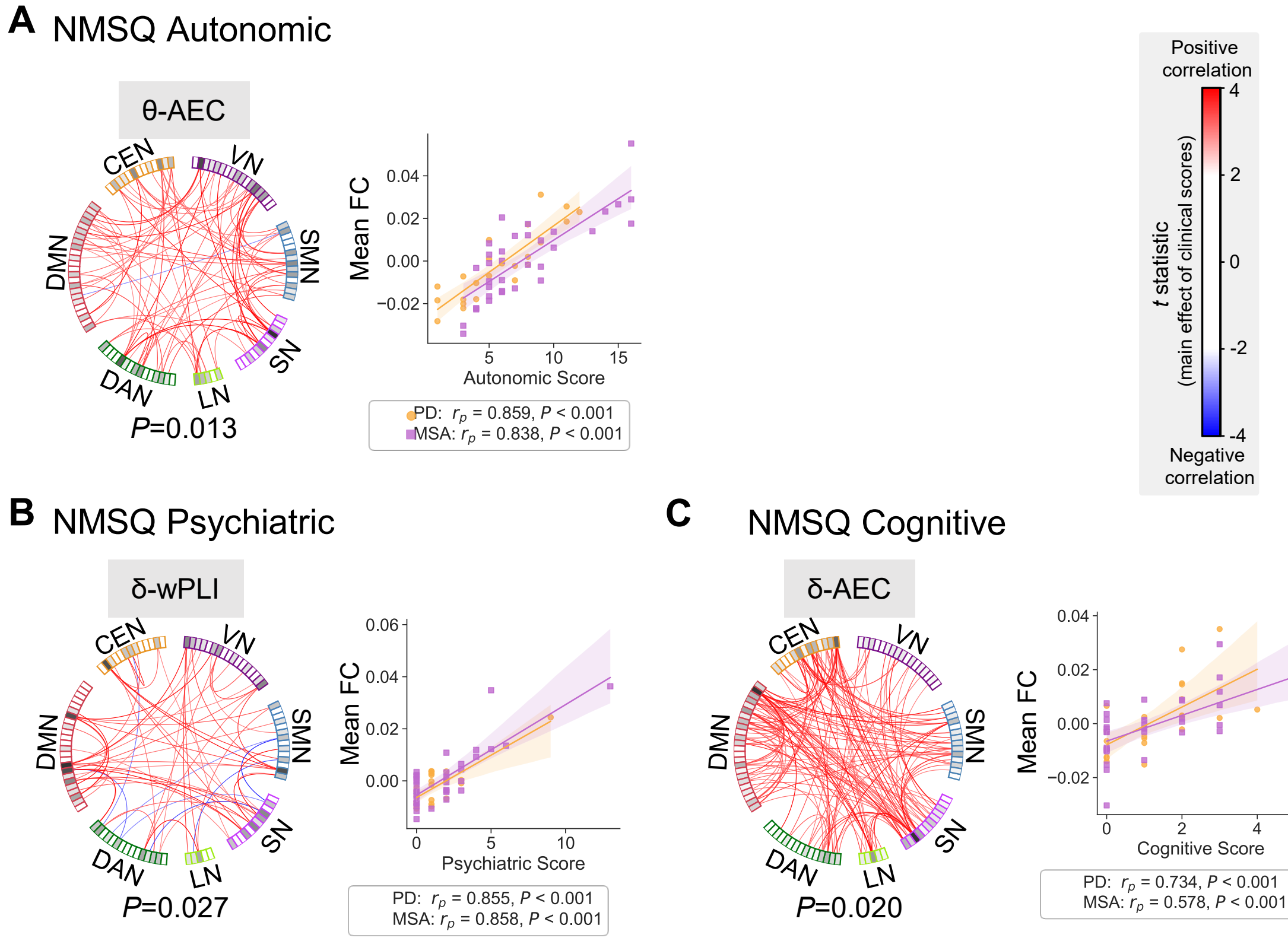

Supplementary Fig. S3 Shared functional network correlates of non-motor symptoms in PD and MSA. (A–C) Main effects of clinical scores on functional connectivity for NMSQ autonomic (A), psychiatric (B), and cognitive (C) sub-scores. For each panel, brain networks (top) display the subnetworks identified via NBS testing for the shared association between symptom severity and connectivity across both disease groups. Edge colors indicate the main effect *t*-statistic for the clinical score. Scatter plots (bottom) illustrate the relationship between the extracted mean subnetwork connectivity and the corresponding clinical score for the PD and MSA groups. To isolate the effect of symptom severity from baseline disease differences, statistical significance of the subnetworks was determined using general linear models controlling for diagnosis and nuisance covariates (age, gender, and recording device). Plotted functional connectivity values on the y-axis represent adjusted residuals from these models. Partial correlation coefficients (r_p) are provided descriptively to demonstrate the consistent, parallel clinical associations within each specific diagnostic group. Note that for all clinical assessments, higher scores indicate greater symptom severity.

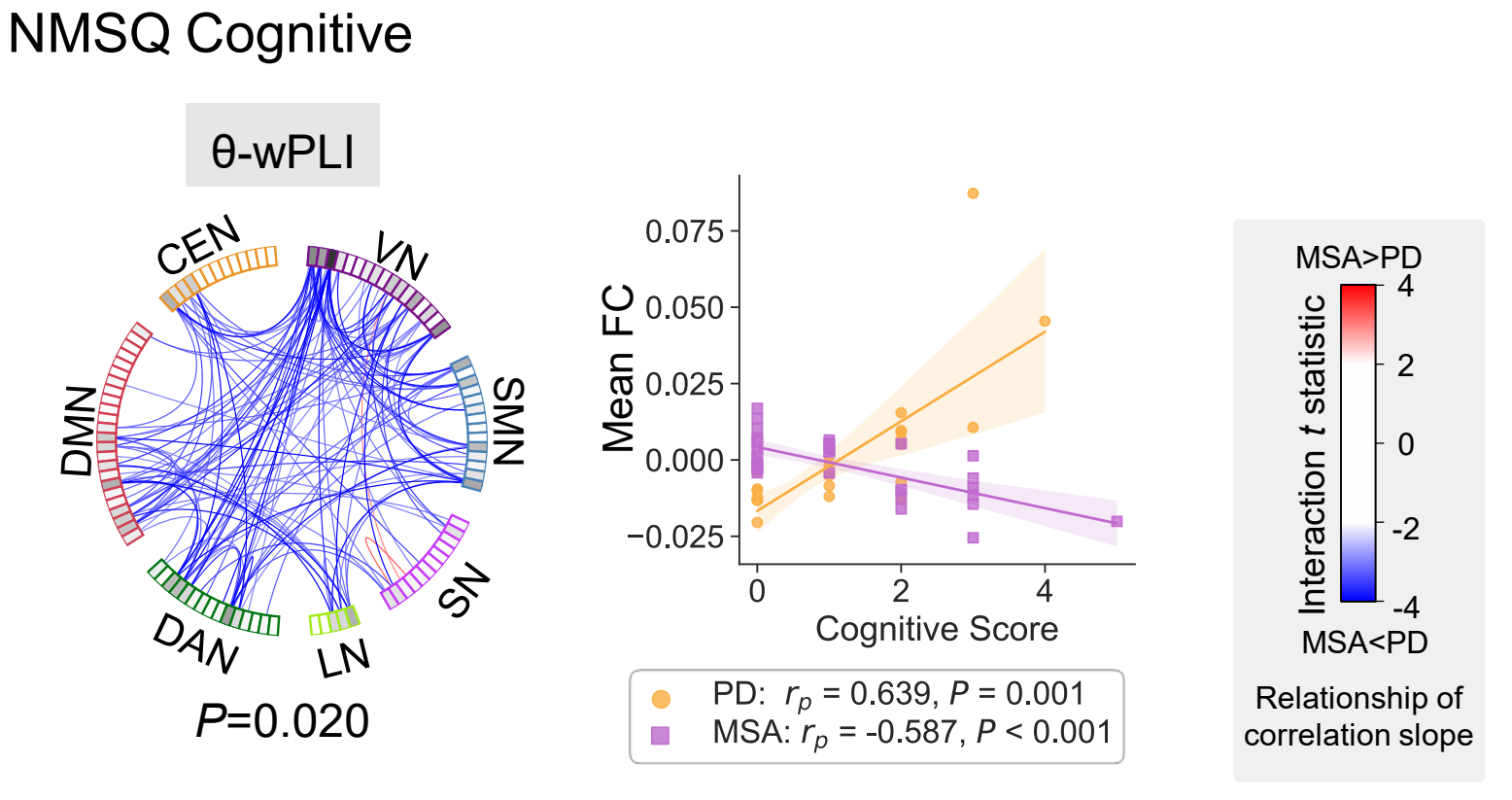

Supplementary Fig. S4 Divergent functional network correlates of non-motor symptoms in PD and MSA. Group × Score interaction effects for functional connectivity and the NMSQ cognitive sub-score is illustrated. Brain networks (left) display the subnetworks identified via NBS interaction testing. Edge colors indicate *t*-statistics for the interaction term: warm colors denote connections where the association between symptom severity and connectivity is significantly more positive in MSA compared to PD, whereas cool colors denote a more positive association in PD. Statistical significance was determined using the NBS procedure, with edge-level interaction *t*-statistics derived from general linear models controlling for nuisance covariates (age, gender, and recording device). Scatter plots (right) illustrate the relationship between the extracted mean subnetwork connectivity and the corresponding clinical score for the PD and MSA groups. Plotted mean FC values are residuals adjusted for nuisance covariates. While NBS provided the primary inferential framework for the interaction effect, partial correlation coefficients (r_p) are provided descriptively to visualize the group-specific clinical associations. Note that higher scores indicate greater symptom severity.

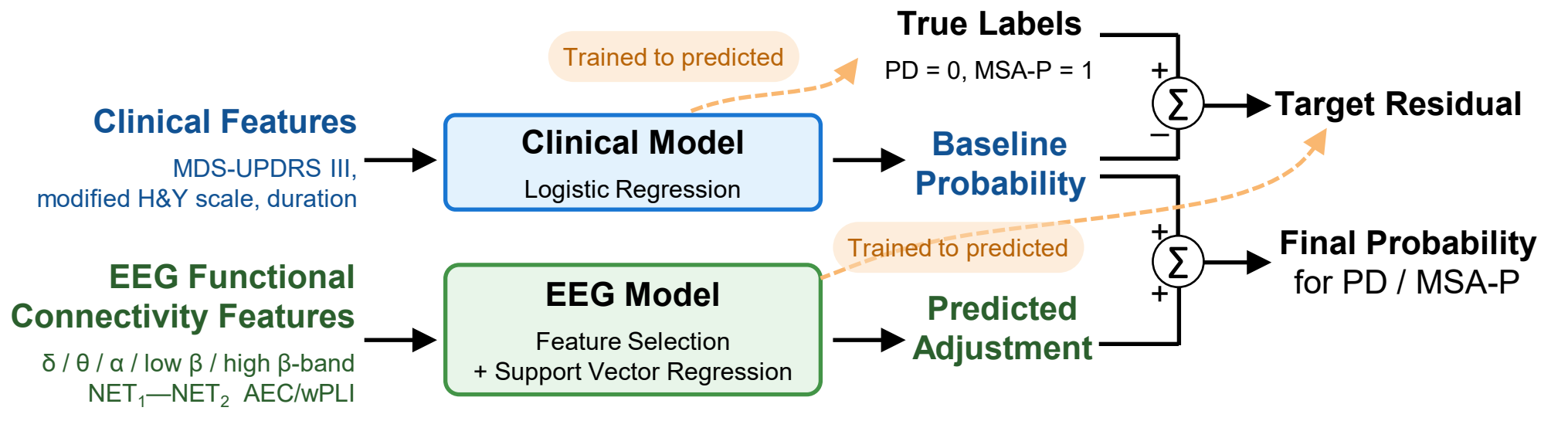

Supplementary Fig. S5 Workflow of the target residual learning approach for PD versus MSA-P classification using clinical and EEG functional connectivity features. A base clinical model (logistic regression) first generates a baseline predicted probability using clinical features. The target residual is calculated as the difference between the true clinical label and the baseline predicted probability. EEG functional connectivity features are then used to train an EEG model (support vector regressor) to predict this residual error. The final predicted probability is computed by adding the EEG-derived adjustment to the baseline clinical probability.
